# Just Sinus Bradycardia or Something More Serious?

**DOI:** 10.1155/2013/736164

**Published:** 2013-02-11

**Authors:** Kelly R. Egan, J. Carter Ralphe, Larry Weinhaus, Kathleen R. Maginot

**Affiliations:** ^1^University of Wisconsin School of Medicine and Public Health, 600 Highland Avenue, Madison, WI, USA; ^2^Division of Cardiology, Department of Pediatrics, University of Wisconsin School of Medicine and Public Health, H6/5 Clinical Science Center, 600 Highland Avenue, Madison, WI, USA; ^3^Division of Pediatric Cardiology, Department of Cardiology, Dean Clinic, 700 S. Park Street, Madison, WI 53715, USA

## Abstract

An asymptomatic 5-year-old girl presented with bradycardia during a routine well-child visit. Further evaluation revealed profound sinus bradycardia, exercise-induced bidirectional ventricular tachycardia, and supraventricular tachycardia. An echocardiogram showed heavy trabeculations in the left ventricular myocardium. This patient's presentation suggested catecholaminergic polymorphic ventricular tachycardia and left ventricular noncompaction. Genetic testing revealed mutations in the cardiac ryanodine receptor (RyR2), calsequestron (CASQ2), and titin (TTN). She was effectively treated with beta-blockade to suppress tachyarrhythmias and pacemaker implantation to treat her bradycardia.

## 1. Introduction

While bradycardia is not uncommon in children [1], profound sinus bradycardia is rare and warrants evaluation. Common causes of sinus bradycardia in children include increased vagal tone, hypothyroidism, hypothermia, adrenal insufficiency, and increased intracranial pressure. Inherited arrhythmias and cardiomyopathies may also cause bradycardia. Catecholaminergic polymorphic ventricular tachycardia (CPVT) is a rare inherited disorder. Patients with CPVT typically have structurally normal hearts and manifest with stress- or exercise-induced ventricular arrhythmias that may lead to syncope, seizures, or sudden death. The baseline electrocardiogram (ECG) often appears normal but may show sinus bradycardia. Like CPVT, left ventricular noncompaction (LVNC) is rare. It is a form of cardiomyopathy that is characterized by hypertrabeculated myocardium and may be associated with ventricular dysfunction, chamber dilation, arrhythmias, and structural congenital heart disease [2]. LVNC is an increasingly recognized form of cardiomyopathy that is very heterogeneous in its clinical presentation. Patients may be completely asymptomatic, while others present with congestive heart failure or sudden death. The following case highlights the evaluation and treatment of a young asymptomatic girl who presented with sinus bradycardia and was found to have exercise-induced bidirectional VT and a hypertrabeculated left ventricle (LV).

## 2. Case

A 5-year-old girl presented to her pediatrician's office for a routine well-child visit. She was asymptomatic and developmentally normal. Parents reported that she participated in all activities but was not as active as her siblings. She was taking no medications. She had never been hospitalized or undergone surgery. Her examination was notable only for bradycardia with a heart rate (HR) of 52 beats per minute (bpm). She had normal weight, height, and blood pressure, with no evidence of thyromegaly. Both parents were healthy, as were the girl's older sister and younger brother. There was no family history of congenital heart disease, seizures, syncope, early sudden death, or family members requiring pacemakers or defibrillators.

**Figure 1:**
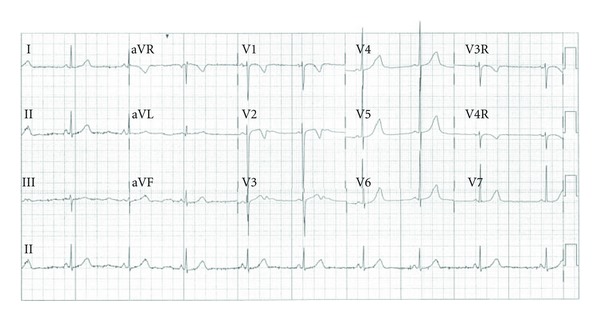
Baseline ECG showing sinus rhythm at 50–60 bpm with normal PR, QRS, and QTc intervals.

**Figure 2:**
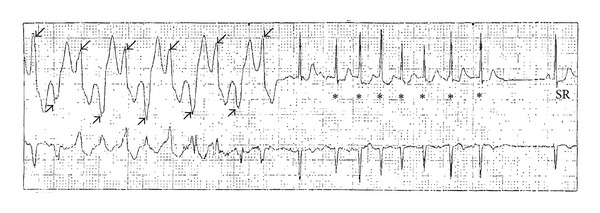
Rhythm strip from Holter monitor showing bidirectional VT, wide QRS complex tachycardia with alternating QRS axis (arrows), followed by nonsustained SVT, narrow QRS complex tachycardia (asterisks), then spontaneous termination to sinus rhythm (SR).

The patient's ECG showed sinus rhythm at 50–60 bpm with normal PR, QRS, and corrected QT (QTc) intervals (Figure 1). Due to the bradycardia, a Holter monitor was performed to evaluate her HR variability. The monitor showed sinus bradycardia with an average HR of 59 bpm and a minimum of 37 BPM. There was no atrioventricular (AV) conduction delay, and repolarization appeared normal. At rates greater than 110–120 bpm, there were frequent polymorphic premature ventricular contractions (PVCs) and bigeminy with runs of nonsustained bidirectional ventricular tachycardia (VT) at 211 BPM, suspicious for a clinical diagnosis of CPVT. Additionally, brief runs of a supraventricular tachycardia (SVT) at a rate of 220 bpm were also noted (Figure 2). The parents reported that their daughter was active at these times and free of any symptoms.

**Figure 3:**
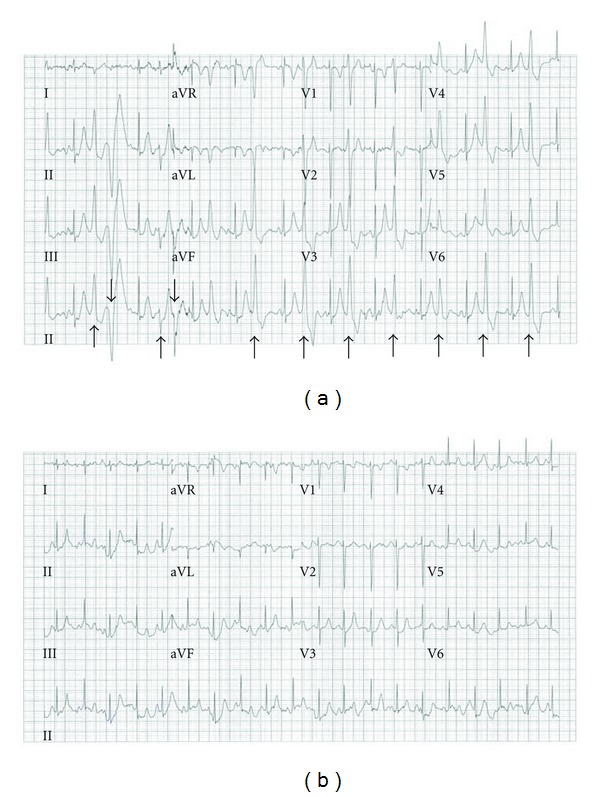
Exercise Treadmill Test. (a) Peak-exercise ECG on initial ETT, prior to antiarrhythmic medications, showing polymorphic PVCs and bigeminy (arrows). (b) Peak-exercise ECG on ETT during esmolol infusion, showing sinus rhythm with suppression of PVCs.

An echocardiogram performed to assess cardiac anatomy and function revealed an overall normal appearing heart with normal LV chamber size and systolic function, but with heavy trabeculations in the LV apex, suggestive of LVNC. Cardiac magnetic resonance imaging showed similar noncompacted myocardium in the LV apex with normal LV chamber size and function and no other abnormalities. Initial laboratory testing including basic metabolic profile, inflammatory markers, complete blood count, and liver and thyroid function tests were within normal limits. The patient underwent an exercise treadmill test (ETT) to assess for inducible arrhythmias in a controlled setting. She had sinus rhythm at 55–70 bpm at rest and developed polymorphic PVCs and bigeminy at HRs greater than 110 bpm (Figure 3(a)). The ETT was terminated at 7 minutes (min) due to the complex ventricular ectopy, although the girl had no symptoms. The PVCs dissipated by 1 min of recovery, as her HR dropped below 100 bpm.

Beta-blocker therapy was initially considered to prevent the tachyarrhythmias, but due to her profound baseline bradycardia, a class 1C sodium channel blocker antiarrhythmic medication (flecainide) was started. A repeat ETT showed no reduction of PVCs during exercise. Therefore, the flecainide was discontinued and a beta-blocker trial was started with esmolol (infusion rate of 250 mcg/kg/min). This short-acting intravenous (IV) beta-blocker was chosen, so that the medication could be discontinued immediately if her basleine bradycardia was exacerbated or the beta-blocker resulted in hemodynamic compromise. A repeat ETT showed resting sinus rates of 50–60 bpm and a peak HR of 118 bpm at 9.5 min of exercise. Rare PVCs were noted with exercise and suppressed completely at peak HRs (Figure 3(b)). Due to potential exacerbation of her underlying bradycardia with beta-blocker therapy, a dual chamber epicardial pacemaker was implanted, and she was started on a long-acting oral *β*-blocker, nadolol (1.0 mg/kg/day). Immediately postoperatively, she had intermittent PVCs and mild hypertension that resolved by increasing nadolol to 2.0 mg/kg/day. Prior to pacemaker implantation, there was a lengthy discussion regarding her arrhythmia substrate and possible implantable cardioverter defibrillator (ICD) placement for primary prevention. Since our patient had no history of syncope and appeared to have an excellent response to beta-blockade, the decision was made against ICD implantation for primary prevention.

Repeat ETT, performed 6 weeks after pacemaker implantation, showed a resting sinus rate of 70 bpm and peak HR of 117 bpm at 10 min of exercise. Rare single monomorphic PVCs noted with exercise suppressed completely at peak HRs. She reported mild fatigue during daily activities, and her pacemaker was reprogrammed on for rate response to allow more physiology heart rate with daily activities. At her 5-month followup she had improvement in her fatigue and no palpitations or syncope. She continued on nadolol with exercise restrictions. Her Holter monitor showed sinus alternating with atrial pacing with good beta-blockade effect and no ventricular arrhythmias. Her echocardiogram was unchanged.

Candidate gene testing was performed due to her history of arrhythmias. A long QT syndrome panel revealed no disease-causing mutations. A pan cardiomyopathy microarray designed to identify mutations in genes associated with cardiomyopathy and CPVT revealed three mutations: ryanodine receptor (RyR2) Arg169Gln, calsequestron (CASQ2) Asp398del, and titin (TTN) Lys4455Arg. These were all single nucleotide changes resulting in missense mutations.

## 3. Discussion

This asymptomatic 5-year-old girl presented with bradycardia and heart rates less than the first percentile for age [3]. Her ECG showed sinus bradycardia with no AV conduction delay and normal QTc. Ventricular arrhythmias with bidirectional ventricular tachycardia were present during exertional activities. Her echocardiogram and cardiac MRI suggested LVNC with normal LV size and function. The results of genetics testing revealed missense mutations in RyR2, CASQ2, and TTN. Bidirectional VT is a rare form of VT characterized by beat-to-beat alternating QRS morphologies within the same ECG lead. Exercise-induced bidirectional VT has been described in rare forms of long QT syndrome, LQT5 and LQT7 (Andersen-Tawil syndrome), and both of which may have a resting QTc within normal limits [4].

Exercise-induced bidirectional VT has also been reported in CPVT. Clinical cases of CPVT were described as early as 1975 [5]. Later the triad of exercise- or emotion-induced severe ventricular tachyarrhythmias, a typical pattern of bidirectional ventricular tachycardia with a normal resting ECG, and a structurally normal heart was reported [6]. Our patient's clinical arrhythmias also included resting sinus bradycardia and exercise-induced atrial arrhythmias that have been described previously in CPVT patients [7–9]. Although the average age of onset of symptoms in patients with CPVT is 7–9 years, life-threatening events have been reported in considerably younger patients and also implicated in cases of sudden infant death syndrome [10]. The patient's parents had reported that their daughter had mildly reduced stamina compared to her siblings and peers. It is likely that this girl's mild exercise intolerance was due to episodes of VT, resulting in diminished cardiac output, that resolved spontaneously when she discontinued exertion.

If left untreated, CPVT has a high morbidity and mortality. Approximately 30% of those affected experience at least one cardiac arrest, and up to 80% have syncope [11]. Beta-blockade is the initial treatment for CPVT. However, flecainide has also been shown to be an effective treatment [12, 13]. We initially chose flecainide in our patient due to her profound baseline bradycardia that would have been exacerbated by beta-blockade. However, flecainide did not appear to suppress her exercise-induced ventricular ectopy. Although technically challenging, this 5-year-old patient completed an exercise treadmill test while on an intravenous infusion of esmolol to assess efficacy of beta-blocker therapy. We chose a short-acting beta-blocker infusion initially in order to have the ability to discontinue the antiarrhythmic medication quickly if symptomatic bradycardia or hypotension occurred. After proving efficacy of arrhythmia suppression with IV beta-blockade, the patient was changed to a long-acting beta-blocker (nadolol), and a pacemaker was implanted due to her baseline bradycardia. Prior to this, there had been a lengthy discussion regarding the risks and benefits of implantable cardioverter defibrillator. Transvenous ICD implantation is difficult in younger patients due to size constraints and potential for device erosion and venous occlusion. Subcutaneous arrays and endocardial leads placed epicardially or subcutaneously have been used successfully, but these lead positions tend to have higher defibrillation thresholds, and battery longevity can be reduced. ICD implantation in children is associated with inappropriate shock, lead malfunction, and patient depression [14–17].

Mutations in both RyR2 and CASQ2, two genes that regulate intracellular calcium metabolism, have been associated with CPVT. RyR2 mutations are inherited in an autosomal dominant fashion and account for 50–55% of genotype-positive CPVT cases [18, 19]. Mutations in CASQ2, autosomal recessive inheritance, account for 1-2% of the CPVT mutations [20]. Interestingly, both RyR2 and CASQ2 mutations may be responsible for cardiomyopathies [21, 22]. Our patient's RyR2 mutation (Arg169Gln) is a single nucleotide substitution resulting in a missense mutation. This mutation has been described previously in a patient with clinical CPVT [23], and it is located in a conserved domain of the RyR2 protein that may be critical for protein interactions.

Interestingly, our patient's CASQ2 mutation had been reported in two patients with LVNC but did not segregate with the clinical disease in the families described. In addition, this mutation causes an in-frame deletion of the second to the last amino acid of the CASQ2 protein and was not considered pathologic.

Although our patient initially had exercise-induced arrhythmias that were consistent with CPVT, she did not have a structurally normal heart. Her heavily trabeculated LV suggested LVNC cardiomyopathy with preserved LV function and no chamber dilation. LVNC is a rare form of cardiomyopathy, previously called spongy myocardium. Based on our current understanding of the pathophysiology of this entity, heart failure and life-threatening arrhythmias appear to be the most significant clinical manifestation. Our patient was negative for known LVNC-causing mutations. She was positive for a TTN mutation (Lys4455Arg). TTN encodes the large sarcomeric protein titin, and TTN mutations have been implicated in cardiac and skeletal myopathies. TTN truncation mutations are a common cause of dilated cardiomyopathy [24]. However, our patient's TTN mutation was not a truncation mutation, and it is uncertain if this mutation plays a role in her myocardial noncompaction.

## 4. Conclusion

This is a unique presentation of bradycardia, exercise-induced atrial and ventricular arrhythmias, and abnormal myocardium in a young and relatively asymptomatic girl. Her tachyarrhythmias were suppressed with beta-blocker therapy, and bradycardia was treated with pacemaker implantation. Mutations in RYR2, CASQ1, and TTN were discovered, and it is uncertain if one or more of these mutations resulted in the phenotype of bradycardia, exercise-induced bidirectional VT, SVT, and LVNC. There is certainly the possibility that a primary mutation may be modified by other mutations found in this young girl. The role of genetic defects and their modifying genes in the manifestation of cardiomyopathies and inherited arrhythmias is an area of intense investigation. Further cardiac evaluation and genetic testing of family members may be helpful in understanding the spectrum of this disease. Unraveling the complex interplay between the multiple mutations in critical cardiac genes found in this patient may provide further understanding of the etiology of her mixed phenotype of arrhythmias and structural heart disease.
